# Supplementary material for: Effect of DSS-Induced Ulcerative Colitis and Butyrate on the Cytochrome P450 2A5: Contribution of the Microbiome
Source: Int J Mol Sci. 2022 Oct 1;23(19):11627. doi: 10.3390/ijms231911627 (PMC9569822; doi:10.3390/ijms231911627)
Supplement: Supplementary file 1 [file ijms-23-11627-s001.zip › ijms-1916776-supplementary.pdf]

Effect of DSS-Induced Ulcerative Colitis and Butyrate on the Cytochrome P450 2A5: Contribution of the Microbiome

Stefan Satka, Veronika Frybortova, Iveta Zapletalova, Pavel Anzenbacher, Eva Anzenbacherova, Hana Kozakova, Dagmar Srutkova, Tomas Hudcovic and Lenka Jourova

Supplementary Data

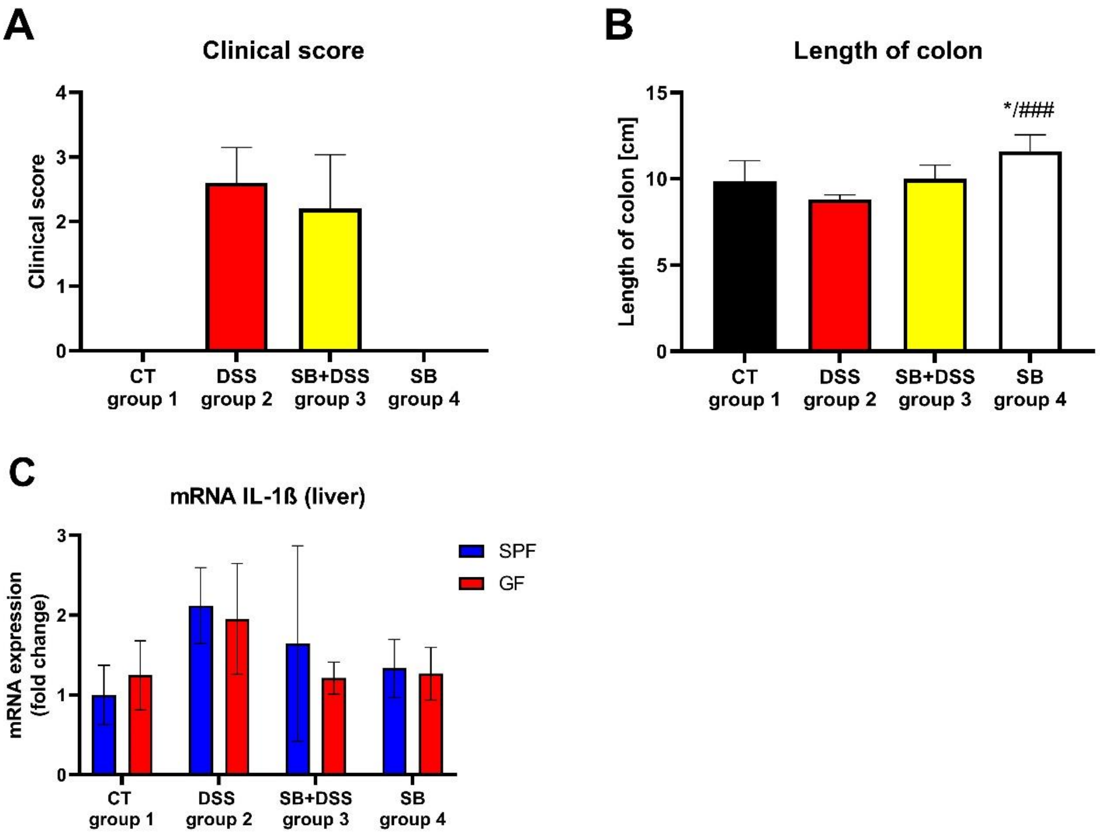

Figure S1. Impact of butyrate pre-treatment on clinical symptoms length of colon and gene expression of IL-1β in the dextran sodium sulfate (DSS) mouse model of ulcerative colitis. **(A)** Occurrence of diarrhea and rectal bleeding in germ-free (GF) mice was summarized in the clinical score. **(B)** Shortening of colon length in GF mice. (C) Gene expression of IL-1β in liver of specific-pathogen-free (SPF) mice and GF mice. Data in graphs are expressed as mean ± SD. One-way ANOVA with Tukey's multiple post hoc test was used for comparison of experimental groups to CT controls (group 1: $*p < 0.05$) or to DSS-controls (group 2: $###p < 0.001$). **CT** (group 1): drinking water for 1 week; **DSS** (group 2): 1 week 2.5% DSS in drinking water; **SB+DSS** (group 3): 0.5% sodium butyrate (SB) in drinking water for 2 weeks followed by a week of 2.5% DSS in drinking water; **SB** (group 4): 0.5% SB in drinking water for 2 weeks.
